# Spatial-Temporal Analysis of Environmental Data of North Beijing District Using Hilbert-Huang Transform

**DOI:** 10.1371/journal.pone.0167662

**Published:** 2016-12-09

**Authors:** Yu Xiang, Xuezhi Wang, Lihua He, Wenyong Wang, William Moran

**Affiliations:** 1 School of Computer Science and Engineering, University of Electronic Science and Technology of China, Chengdu, Sichuan, China; 2 School of Electrical & Computer Engineering, RMIT University, Melbourne, Australia; West Virginia University, UNITED STATES

## Abstract

Temperature, solar radiation and water are major important variables in ecosystem models which are measurable via wireless sensor networks (WSN). Effective data analysis is necessary to extract significant spatial and temporal information. In this work, information regarding the long term variation of seasonal field environment conditions is explored using Hilbert-Huang transform (HHT) based analysis on the wireless sensor network data collection. The data collection network, consisting of 36 wireless nodes, covers an area of 100 square kilometres in Yanqing, the northwest of Beijing CBD, in China and data collection involves environmental parameter observations taken over a period of three months in 2011. The analysis used the empirical mode decomposition (EMD/EEMD) to break a time sequence of data down to a finite set of intrinsic mode functions (IMFs). Both spatial and temporal properties of data explored by HHT analysis are demonstrated. Our research shows potential for better understanding the spatial-temporal relationships among environmental parameters using WSN and HHT.

## 1. Introduction

Temperature, solar radiation and water are considered to be the most important microclimatic drivers that modulate the magnitude and frequency of carbon fluxes [1] and have always been important variables in ecosystem models [2, 3]. Adequate understanding of these field environment data as functions of space and time is necessary for developing empirical models of soil-vegetation-atmosphere transfer [4]; also it could help in expert system design of precision agriculture [5] and quantified the uncertainty caused by *in-situ* method for satellite remote sensing validation and calibration [6]. Given measurement data, it is still a challenge to understand and characterize the observations across multiple space-time scales.

Previous studies have shown that each single field environment parameter listed above (land surface temperature (LST), soil moisture or solar radiation) has strong spatial-temporal variability and the patterns of these data are nonlinear and nonstationary. Western et al. pointed out that the spatial and temporal soil moisture variability are largely determined by factors like soil type, vegetation and atmospheric forces, which interact in nonlinear way [7] and are deemed to be nonlinear processes [8]. Solar radiation is the most important source of energy required for plant growth. The spatial-temporal variation characteristics of photosynthetically active radiation (PAR) in both site-scale and regional-scale are exploited by Hu et al. and Zhu et al. in [9, 10]. Barnhart et al. consider the variation of global temperature as a random nonlinear process and analysed its cycles in different time scales [11]. Ouyang et al. investigated on PAR and land surface temperature data in northwest Ohio, USA. They claim that PAR and land surface temperature data are highly correlated because of their similar diel and annual cycles [12]. However, little effort has been made for a complete examination of these three parameters (LST, soil moisture and PAR) in a same region and the spatial-temporal relationships among these three parameters.

Wireless sensor networks (WSN) for data collection in this work is configured by dense deployment of several different kinds of environmental sensor nodes which continuously take measurement on field environment in various spots and pre-processed in real time. Although other methods, such as meteorological station and satellite remote sensing, may be used to observe environment parameters, WSN has many advantages over conventional solutions, such as lower cost, long-time monitoring, scalability, accuracy, and ease of deployment. These enable WSN to be applied for environmental and agricultural applications [13]. For example, Langendoen et al. made the first large-scale experiment (LOFAR-agro) using WSN in precision agriculture in the Netherlands [14]. Morais et al. implemented WSN to collect climate data with soil moisture for smart irrigation in Portugal [15]. Zhang et al. utilized sensor network to monitor land surface temperature, humidity, ambient light, soil moisture and temperature that helped them in analysing the current state of plant nursery [16].

As mentioned above, patterns of these environment data are proved to be nonlinear and nonstationary. Traditional data analysis methods such as short-time Fourier transform or continuous wavelet transform use an a priori established basis. But if the data are nonlinear and nonstationary, these transforms may not lead to physically meaningful results. So we need to have an adaptive basis [17]. Some method to analysis the temporal-spatial related time series are introduced in [18–21]. A relatively new-developed method, the Hilbert-Huang Transform (HHT), seems to find a better way for nonlinear and nonstationary data analysis, especially for time-frequency-energy representations [17]. By now this method has been already applied in the field of atmospheric, hydrological and ecological sciences [11, 22, 23, 24].

In this paper, a HHT based data analysis technique is adopted to decompose environmental data into physically meaningful components. This enables a statistical analysis to be carried out, which provides great insights into the spatial-temporal relationships between land surface temperature, soil moisture and solar radiation for the environment of the area covered by the WSN. Data collection that consists of more than 200,000 environmental measurements is obtained in 2011. Apart from a limited analysis report, this is the first time that we use the HHT and statistical data analysis tools to explore new information from this data set.

The rest of this paper is arranged as follows. First, we describe the wireless environment sensor network which we deployed for data collection in Yanqing, Beijing, China. Second, temperature, soil moisture and solar radiation measurements are decomposed into components in time domain via EMD/EEMD technique, the component level data is then further processed by HSA to get time-frequency-energy representations. Then the correlation parameters among the decomposed components are calculated, compared and analysed. Analysis results are illustrated and discussed in Section 3 which is followed by the Conclusions.

## 2. Materials and methods

### 2.1 Hilbert-Huang Transform (HHT)

Hilbert-Huang Transform consists of two parts: empirical mode decomposition (EMD) and Hilbert spectral analysis (HSA). EMD separates input data sets into finite and often small number of intrinsic mode functions (IMFs) at different scales plus a residue, each IMF with its average circle, represents decomposed characteristic of the original time series at this time scale and residue shows the tendency of the original time series; HSA on the IMFs provides a possible extraction of instantaneous frequencies and amplitudes (IF and IA), with which a time-frequency-energy distribution model could be built [24].

#### 2.1.1 EMD and Ensemble EMD (EEMD)

The original EMD method [17] is based on the assumption that the underlying data consists of different intrinsic modes of oscillations. Each of these oscillatory modes is represented by an intrinsic mode function (IMF) with variable amplitude and frequency as functions of time. Let *x(t)* be the input data, the EMD is given by

$$x(t)=\sum_{i=1}^{n}{i=IMF}_{i}(t)+r_{n}(t)$$ (1)

Where *r*_*n*_*(t)* is a monotonic function and represents the residue of *x(t)*. Each *IMF*_*i*_ has the following properties: (1) the number of extreme points (maxima or minima) is within 1 of the number of zero-crossings in the entire time series; (2) at any data location, the mean value of the upper envelope defined by the local maxima or the lower envelope defined by the local minima is zero.

**Fig 1:**
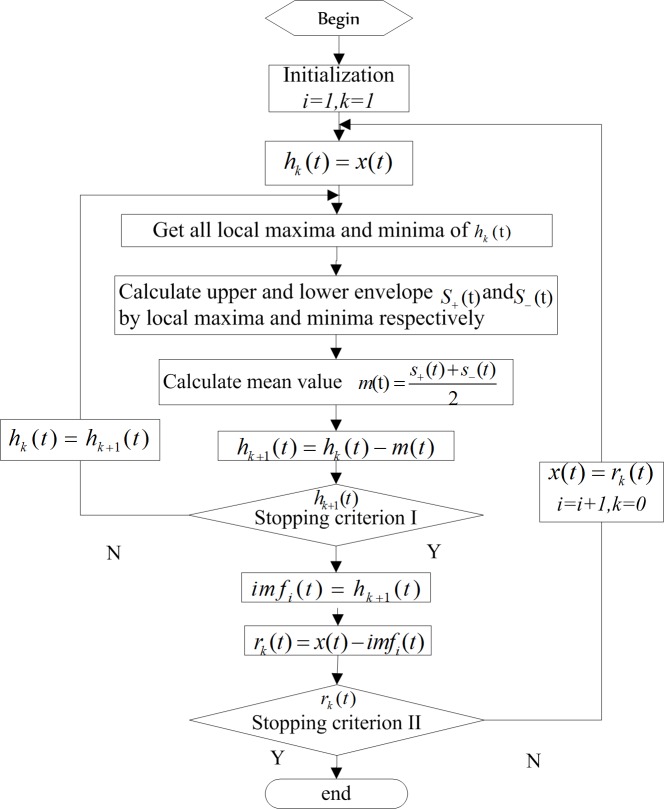
EMD algorithm

In practise, EMD algorithm can be implemented through a sifting process which include two iterations: one inner loop, which uses local extrema and stopping criterion I to generate a single IMF, and an outer loop, which stops when the residue, *r*_*n*_*(t)*, becomes a monotonic function from which no more IMF can be extracted (i.e. stopping criterion II). The EMD algorithm is shown in Fig 1 below.

As shown in Fig 1, different versions for stopping criterion I were proposed [25]. Huang et al. have shown that fixing the sifting number (i.e. the iteration number of the inner loop) to a low number in the EMD is the key to keep the EMD temporally “local”, that is, the upper and lower envelops go through all of the local maxima and local minima respectively [17]. Wu et al. gave an empirical guide in [26] and concluded that if we wish the EMD to be similar to an adaptive dyadic filter bank, S should be around 10. Stoppage criterion II could be represented by the total number of IMFs of an input data set. For a time series with length N, the commonly used in EMD algorithm usually results in close to but no more than log2N IMFs [26].

In order to avoid a serious “mode mixing” problem, which manifests itself in IMFs consisting of oscillations of dramatically disparate scales in EMD, Wu and Huang, inspired by a noise-assisted data analysis (NADA) method, proposed an improved algorithm of EMD–Ensemble EMD (EEMD) [27].

EEMD algorithm is described as follows:

1. Add a white noise series to the input data;
2. Decompose the data with added a white noise into IMFs;
3. Repeat step 1 and 2 by N (ensemble) times, each time with different white noise;
4. Obtain the means of corresponding IMFs as the final decomposition result.

Up to now, EEMD has been used in a few amounts of environmental studies such as [28] and [29].

#### 2.1.2Hilbert spectral analysis (HSA)

After the IMFs are obtained, HSA is applied to each IMF component, and instantaneous amplitude and frequency are computed. Most popular way to get the HSA results is by using the Hilbert transform [17]. Bedrosian and Nuttall found out that one cannot always obtain a physically meaningful IF by the traditional methods, and some conditions must be satisfied [20]. Huang et al. proposed a normalization scheme called normalized Hilbert transform (NHT) combined with direct quadrature (DQ) to compute physically meaningful IF. NHT includes an iteration based on cubic spline fitting to decompose AM and FM empirically. NHT and DQ combined algorithm is described as follows:

1. For the given IMF data *x(t)*, find all the local maxima of the absolute value of the data. Next, connect all these maxima points with a cubic spline curve to form an empiric envelope of the data, described as *e*_*1*_*(t)*.
2. Use *e*_*1*_*(t)* to normalize *x(t)*, that is, $y_{1}(t)=\frac{x(t)}{e_{1}(t)}$ with *y*_*1*_*(t)* as the normalized data. Note that the normalized data may still have amplitudes higher than 1 occasionally [24]. Back to step1 with *x(t)* replaced by *y*_*1*_*(t)* in order to remove any results of this type. Then after n iterations, we get $y_{2}(t)=\frac{y_{1}(t)}{e_{2}(t)}$ … $y_{n}(t)=\frac{y_{n-1}(t)}{e_{n}(t)}$. When all the values in *y*_*n*_*(t)* are less than or equal to 1, the normalization is finished and turn to Step 3. We need to find its envelope *a(t)*, and carrier *cos θ(t)*, that *x(t) = a(t)cosθ(t) = a(t)F(t)*. The FM part of the *x(t)*, is given by
  $$y_{n}(t)=cos\;\theta (t)=F(t)$$ (2)
  Then the AM part, *a(t)*, is defined as
  $$a(t)=\frac{x(t)}{F(t)}=e_{1}(t)e_{2}(t)\cdots e_{n}(t)$$ (3)
3. According to (2), compute its quadrature as
  $$sin\;\theta (t)=\sqrt{1-F^{2}(t)}$$ (4)
  Then get the phase function from
  $$\theta (t)={tan}^{-1}\frac{F(t)}{\sqrt{1-F^{2}(t)}}$$ (5)
4. Calculate instantaneous frequency (IF) as
  $$\omega (t)=\frac{d\theta (t)}{dt}=\frac{1}{a^{2}}(xy^{\prime }-yx^{\prime })$$ (6)

The method above does not need HT and enables us to get exact IF and IA. The Hilbert energy spectrum could also be defined as the squares of the amplitudes. In the subsequent sections, we will use traditional EMD and EEMD to decompose field environment data into IMFs. Then we will use NHT and DQ to process IMFs to calculate IF and IA. The results will be compared and analysed later.

### 2.2 Data collection with WSN

#### 2.2.1 System overview

**Fig 2:**
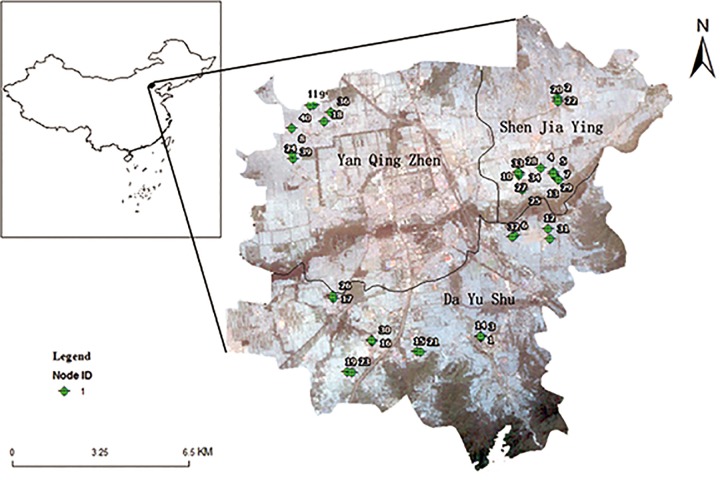
Node location and their associated catchment area

**Fig 3:**
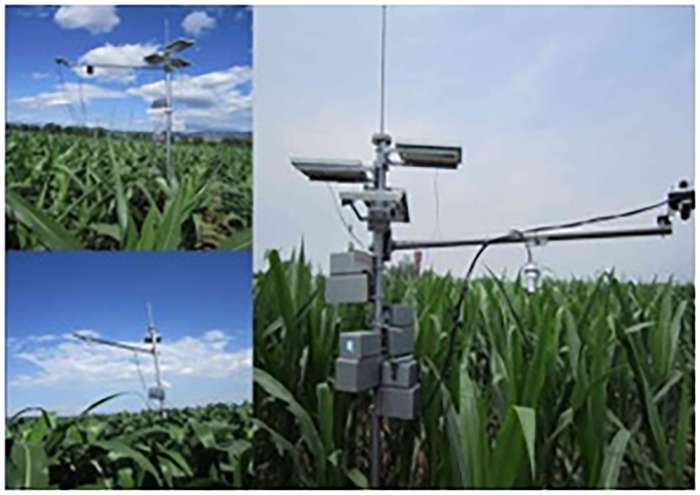
Nodes’ deployment in field

The wireless environment sensor networks (WSN) for data collection in this paper were deployed as a part of the China Next Generation Internet (CNGI) project. This WSN is used to continually monitor crop field environment parameters, including land surface temperature, soil moisture and solar radiation, for the entire growing season. The monitoring field is located in Yanqing County, just outside of the Great Wall in northwest of Beijing CBD and field area is approximately 100 km^2^. Total 36 WSN nodes consisting of 108 sensors were deployed in three groups across 3 rural townships. WSN nodes in each group are further divided into several sub-groups as shown in Fig 2 and Table 1. The wireless sensor node used in this project is named as “Tarax-Node”, which is an IPv6 WSN node designed and implemented in our previous work presented in [30]. Fig 3 shows three of Tarax-Nodes deployed. The system includes two functions: 1) Tarax-Nodes are used to gather and transmit field environment measurements to monitoring centre with local and long-distance communication; and 2) the monitoring centre processes the data and provides analysis and illustrations tools to users [31].

**Table 1:** WSN group and location.

Group (Township)	Sub-group (Village)	Node’s Number	Sub-group (Village)	Node’s Number
1 (Da Yu Shu)	1	16,30	5	17,26
	2	19,23	6	6,32
	3	1,3,14	7	12,31
	4	15,21	—	—
2 (Shen Jia Ying)	1	25,34	4	7,13
	2	10,27,28,33	5	4
	3	5,29	6	2,20,22
3 (Yan Qing Zhen)	1	9,11	3	18
	2	8,24,39	4	36

#### 2.2.2 Data Sets

We installed three types of sensors on each of WSN nodes: infrared thermometer (TPT300V/2-B), soil moisture sensor (SWR2) and photonic sensor (SQ-222/SQ-225). The instrumentation was completed in June 2011, with 36 infrared thermometers, 36 soil moisture sensors and 36 photonic sensors. From July to September, more than 230,000 sensor measurements were taken with measurement interval 1 hour and they were stored in a central database.

**Fig 4:**
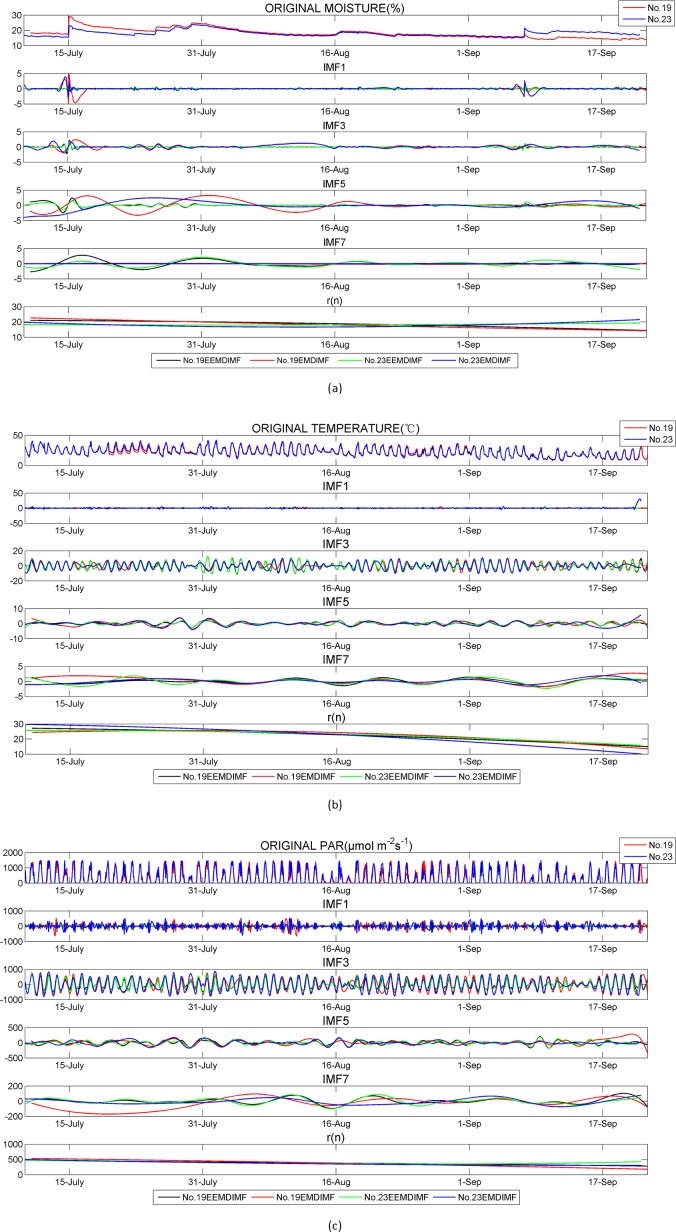
Data and decomposition

In Fig 4, we plot the sensor measurements taken by nodes No. 19 and No.23 (see Table 1). The first plot in Fig 4(A) is the soil moisture measurements in [v]/[v], the first plot in Fig 4(B) is the canopy temperature measurements in centigrade, and the first plot in Fig 4(C) shows pressure measurements in *μ*mol m^-2^s^-1^.

## 3. Results and Discussion

### 3.1 Data Decomposition and HSA

All sensor measurements from each WSN nodes were decomposed separately for each of environmental parameters (temperature, moisture and PAR). The HHT on data was carried out using the MATLAB program which was coded based on the source code available at http://rcada.ncu.edu.tw/. The iteration stopping criteria I and II used in our algorithm are the same as that described in Section 2.1. In EEMD, the standard deviation of added white noise is set to 0.4, and ensemble time NE is 100. The results of the decomposition of data collected from group 1–2 are shown in Fig 4. Similar results are seen for data collected from all of other groups and thus will not be shown here.

**Fig 5:**
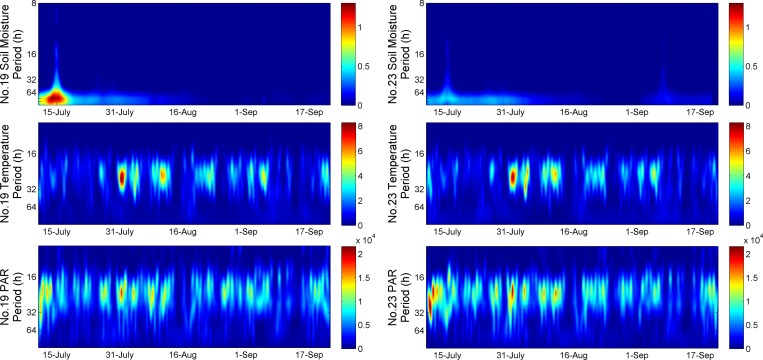
Hilbert Spectrum of No.19 and No.23

Fig 4 indicates that both EMD and EEMD are able to decompose original data into different IMFs with a residue. By visual inspection, it is observed that with EMD method, in many cases of short-time or middle-time scale IMF of temperature, moisture or PAR, the curves of neighbouring IMFs have similar periods. That is due to “mode mixing” problem caused by EMD. While with EEMD, synchronization between neighbouring IMF pairs is greatly improved, thus cause the correlation values of the corresponding IMFs increase. This may be due to the EEMD which helps to isolate the signals of various scales to identify the true time scales of consistent coupled oscillations in the individual IMFs [26]. Thus we use the decomposed results of EEMD in our afterward discussion. The Hilbert spectrum of the corresponding data is shown in Fig 5. The horizontal axis of each plot in Fig 5 displays the time period in hours, and the colours represent energy level (i.e. squared amplitude). In Fig 4(A), we can see two sudden jumps in soil moisture on 15 July and 3 September, respectively. These jumps were caused by heavy rain at those times. The components of short-time and middle-time scales contribute to these sudden moisture increases, as shown in IMF1—IMF_3_ in Fig 4(A). The soil moisture energy spectra in Fig 5 also shows that an energy concentration in those two days clearly. Relatively high energy concentrations for temperature and PAR in some days in July and August also could be found in Fig 5. This means HSA can provide strong localized spectrum information which makes the abnormal values easily to be detected. We could find periodicity and similarity in temperature and PAR from their Hilbert spectrum and we will analyse them in Section 3.2 and 3.3.

In Fig 4(B), the curve of residue *r(n)* reaches its peak in the middle of July, then it slowly decreases between the end of July and September. Since residue *r(n)* represents “trend” of the original data to be decomposed, this curve displays the temperature trend in Yanqing’s summer correctly. The residue curve of PAR in Fig 4(C) also slowly deceases from July to September because of weaker solar radiation.

### 3.2 Cycles

Since each IMF represents different intrinsic modes of oscillation, it is possible to calculate the period for each of IMFs. Table 2 shows the average periods calculated by the time intervals between consecutive zero-crossings on successive waves. In Table 2, M1 in the first row means the average cycle of soil moisture data in group1, as mentioned in Table 1. T1 means the average cycle of temperature data in group1. P1 means the average cycle of PAR data in group1.

**Table 2:** Periods (in hours) of decomposed data series with EEMD.

	M1	M2	M3	T1	T2	T3	P1	P2	P3
IMF_1_	3.2	3.2	3.2	3.3	3.3	3.3	3.4	3.4	3.4
IMF_2_	6.9	6.9	6.9	8.5	8.7	8.7	9.9	9.9	9.9
IMF_3_	16.0	15.1	16.1	**23.6**	**23.6**	**23.8**	23.3	22.9	22.4
IMF_4_	**27.7**	**28.0**	**28.4**	29.1	28.7	28.4	**24.5**	**24.4**	**24.4**
IMF_5_	65.7	72.0	68.7	77.8	74.4	78.9	64.7	66.0	63.2
IMF_6_	167.1	143.8	170.8	173.4	168.0	161.6	119.4	114.4	118.2
IMF_7_	347.5	336.9	364.2	319.3	336.4	343.9	239.2	290.6	272.5
IMF_8_	**672.5**	**739.5**	**690.7**	**821.8**	**753.4**	**740.4**	**608.3**	**638.6**	**564.4**
IMF_9_	1210	1547.2	1478.5	1538.8	1461.2	1360.5	1625.7	1197.3	1179.5

Although the three kinds of field environment parameters (soil moisture, LST and PAR) are physically dissimilar and are measured in different instruments, the decomposed data show nearly identical periods for the lower IMFs such as IMF_1_ to IMF_4_ in all three parameters. In particular, the average periods for both moisture and solar radiation values of IMF_4_ are approximately 24h and for temperature values of IMF_3_ 24h. We highlight those values in bold in Table 2. This indicates that HHT has the ability to extract common scales from original data accurately, which makes it possible to compare the scale-specific temporal relationship on different groups in different locations. We will further analyse the correlation on this one-day circle in Section 3.3 and 3.4.

Moreover, we can see from Table 2 that a nearly 1 month period (around 720 hours) in IMF_8_ but not as accurate as that in IMF_4_. The higher IMFs, which represent the longer periodic oscillations of the original data, have larger differences because there are fewer cycles to average over fluctuations in instantaneous frequencies, as we can find in IMF_7,8,9_.

### 3.3 Temporal Correlation of Different Parameters and IMF Comparisons

All the sensor measurements were decomposed into their IMFs. Then we use Spearman’s rank correlation to analyse the relationship among these IMFs. The Spearman correlation coefficient is “nonparametric”, i.e., it can be obtained without prior knowledge of the joint probability distribution for the data to be compared. A high value of the Spearman correlation coefficient implies a stronger relationship between two data series. The sign of the Spearman correlation indicates the direction of association between *X* (the independent variable) and *Y* (the dependent variable). If *Y* tends to increase when *X* increases, the Spearman correlation coefficient is positive. If *Y* tends to decrease when *X* increases, the Spearman correlation coefficient is negative.

Temporal correlation of our measurements is discovered by two steps using time series of IMFs. First, we calculate the Spearman’s rank cross-correlation coefficients of each IMFs of parameter pairs (T, P), (T, M), (M, P) in and between each subgroup with IMFs combination respectively, then we calculate the average cross-correlation coefficients value of each IMFs combination. Average results of parameter pairs (T, P), (T, M), (M, P) are shown in Table 3, Table 4 and Table 5. Here T represents temperature, P represents PAR and M represents soil moisture.

**Table 3:** Average Cross-correlation Coefficients–(T, P).

	IMF_1_	IMF_2_	IMF_3_	IMF_4_	IMF_5_	IMF_6_	IMF_7_	IMF_8_	IMF_9_
IMF_1_	0.1802	0.0651	-0.0039	-0.0136	-0.008	-0.0026	-0.0001	0.0013	-0.0051
IMF_2_	0.0819	**0.5301**	0.2442	0.0641	0.0162	0.0178	0.0131	0.0143	0.0064
IMF_3_	-0.0082	0.1048	**0.8474**	**0.7889**	-0.0231	-0.0377	-0.0422	-0.0428	-0.0124
IMF_4_	-0.009	-0.0167	0.4722	**0.6965**	0.1533	-0.0004	0.0112	0.0174	0.0098
IMF_5_	0.015	0.0071	-0.0554	0.0591	**0.4737**	0.1676	0.0329	0.0101	0.0253
IMF_6_	0.0021	-0.002	-0.0169	0.0041	0.029	0.0551	0.0935	0.0039	-0.0242
IMF_7_	0.0006	0.0223	-0.066	-0.0042	0.0017	0.1177	**0.5496**	0.3445	0.0982
IMF_8_	0.0009	0.0055	-0.0336	-0.0035	-0.0074	-0.0261	0.1294	**0.4662**	0.2953
IMF_9_	0.0019	0.0099	-0.035	-0.0011	-0.0084	-0.0064	0.0627	0.1554	**0.4424**

**Table 4:** Average Cross-correlation Coefficients–(M, T).

	IMF_1_	IMF_2_	IMF_3_	IMF_4_	IMF_5_	IMF_6_	IMF_7_	IMF_8_	IMF_9_
IMF_1_	-0.007	-0.0002	0.0062	0.0077	0.0058	0.0016	0.008	0.0032	0.0033
IMF_2_	0.0045	-0.0078	0.0089	0.004	0.0054	0.0024	0.003	0.0027	0.0028
IMF_3_	-0.0025	0.0341	**0.208**	0.1397	-0.0036	-0.0085	-0.0069	-0.0026	0.0045
IMF_4_	-0.0013	0.0218	**0.2615**	**0.1937**	0.006	0.0176	0.0167	0.0027	-0.0039
IMF_5_	-0.0044	-0.0019	-0.0085	0.0544	0.0824	0.0262	0.0446	0.0418	0.0014
IMF_6_	-0.0051	-0.0023	-0.0077	-0.0033	0.025	-0.1405	-0.1075	0.0861	0.0458
IMF_7_	0.001	-0.0087	0.0134	-0.0183	0.0272	0.0057	**-0.2395**	-0.355	-0.082
IMF_8_	-0.0021	-0.0015	0.0125	-0.0112	0.0279	-0.075	-0.0578	**-0.2871**	-0.041
IMF_9_	-0.0003	-0.005	0.0086	-0.0036	0.0028	0.0208	-0.0395	-0.2656	**-0.1474**

**Table 5:** Average Cross-correlation Coefficients–(M, P).

	IMF_1_	IMF_2_	IMF_3_	IMF_4_	IMF_5_	IMF_6_	IMF_7_	IMF_8_	IMF_9_
IMF_1_	-0.0044	0	0.0063	0.0039	0.0032	0.0011	0.0048	0.0043	-0.0002
IMF_2_	-0.0027	-0.019	0.001	0.0007	-0.0008	0.0034	-0.0015	-0.0001	0.0005
IMF_3_	-0.01	-0.011	**0.0791**	0.0887	-0.0089	-0.0094	-0.0116	-0.0094	-0.002
IMF_4_	-0.008	0.006	**0.1148**	**0.1615**	0.0304	-0.0238	0.0117	0.0109	-0.0005
IMF_5_	0.0017	0.0032	-0.0186	0.0196	0.0968	0.0812	0.0292	0.0513	0.0065
IMF_6_	0.0028	-0.005	0.003	0.0028	0.0516	0.0677	-0.0647	0.0251	0.0453
IMF_7_	-0.0033	-0.0104	0.036	-0.0017	0.0104	-0.0067	**-0.1721**	-0.2551	-0.1188
IMF_8_	0.0004	-0.0098	0.012	0.0049	0.0167	0.0289	-0.0479	**-0.3245**	0.0307
IMF_9_	-0.0024	0.0011	-0.0073	0.0029	0.0175	0.0282	-0.0458	-0.215	**0.1425**

**Fig 6:**
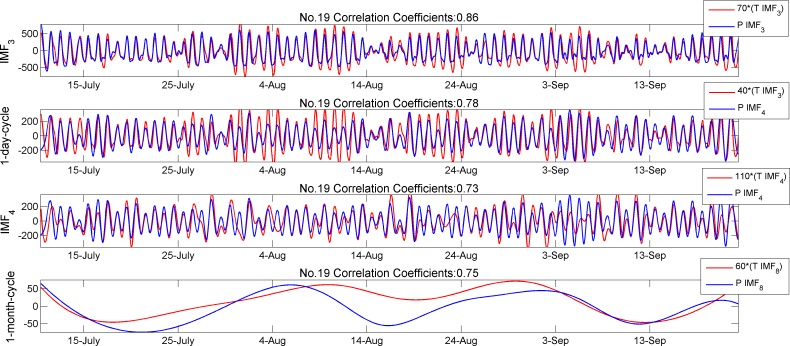
Comparison of IMFs between temperature and PAR

It is not surprising that the IMFs of temperature are highly correlated with those of solar radiation. These values in Table 3 are highlighted in bold and they indicate that higher correlations always exist between the same time scales. The highest value of cross-correlation coefficients 0.85 appears at matrix position (3, 3) and the second highest value 0.79 at (4, 3). Compared with Table 2, we can find out that these highest values appear with time scales for both IMF components are around 24 hours. This implies a very similar variation between PAR and temperature in one-day-cycle. In Fig 6, we plot IMFs of T and P in the subgroup 1–2 (nodes No.19) together for a comparison. Similar results are observed for all other different nodes.

From Fig 6, it is clear that in both approximate 1-day and 1-month cycles the IMF curves of temperature and solar radiation are similar. They arrive at local extrema (positive and negative extreme values) almost at the same time across the entire time axis. The curves here clearly evidence the high correlation coefficient values we calculated in Table 3.

**Fig 7:**
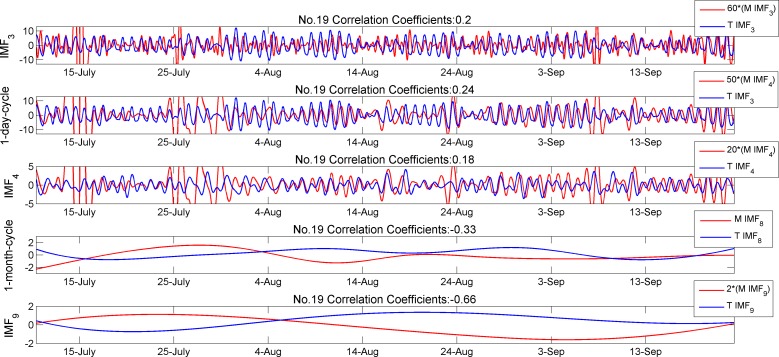
Comparison of IMFs between temperature and soil moisture

**Fig 8:**
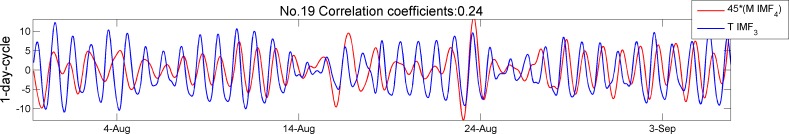
Enlarged view of 1-day cycle

Table 4 demonstrates the correlation matrix of soil moisture and temperature (i.e. (P, T) pairs). Similarly, the correlation coefficient matrix is diagonally dominated. The absolute correlation values around one-day cycle (IMF_3_-IMF_3_, IMF_4_-IMF_3_, IMF_4_-IMF_4_) are smaller than those in the (T, P) pairs, which indicates that soil moisture and air temperature are less correlated compared to that between air temperature and PAR. Fig 7 shows two parameter’s IMF curves of subgroup 1–2 (node No.19). The curves in the first three plots have similar shapes but are different in local extrema and phase shift values across all time periods. An enlarged view of this inconsistent local extrema and phase shift in 1-day cycle IMF curves is shown in Fig 8.

Negative correlation values appear in Table 4 of the long-time scale (i.e. IMF_6_ –IMF_9_) mean that if temperature tends to increase when soil moisture decreases and vice versa. Long-term soil moisture-temperature interactions have only received attention recently in the climate modelling community [32, 33]. Reduction in soil moisture appears to be correlated with a feedback mechanism of evapotranspiration with temperature. Increased temperature leads to a higher vapour pressure deficit and evaporative demand, and thus to a potential increase in evapotranspiration despite the dry conditions, possibly leading to a further decrease in soil moisture [32]. The 4th and 5th plots in Fig 7 show that the two curves differ in phase by 180 degrees and these plots visually highlight why the high negative correlation values appear in Table 4. In addition, in view of the 6th plot in both Fig 4(A) and 4(B), the trend of temperature and soil moisture along with opposite phases can also be observed. Up to now, the research on soil moisture-temperature coupling mainly focused on the global scale. Our results provide insights and thus help us achieve a better understanding the coupling relationship of the measured parameters at local scales.

**Fig 9:**
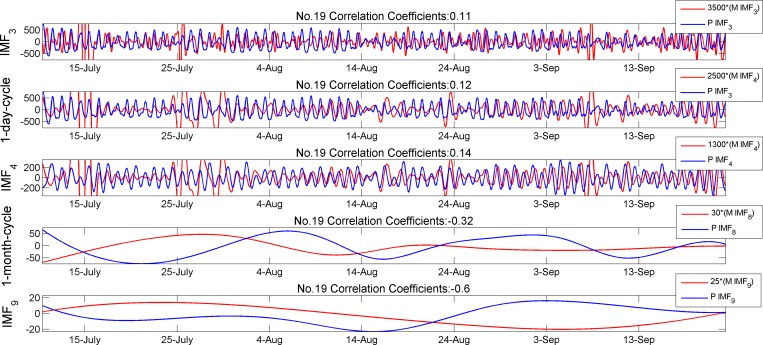
Comparison of IMFs between PAR and soil moisture

**Fig 10:**
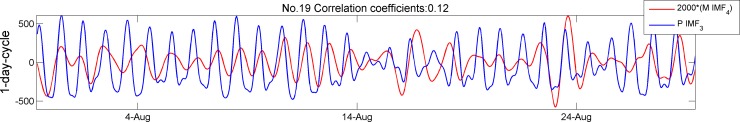
Enlarged view of 1-day cycle

The correlation matrix of soil moisture and PAR (i.e. (M, P) pairs) are shown in Table 5. We can see from Table 3 that a relatively high relationship exists between (T, P) pairs and the relations between (M, T) pairs are also illustrated in Table 4. Thus some degree of correlations between soil moisture and solar radiation are certainly existed and could be observed in Table 5, but no research work so far demonstrates such relationship in literature. We can see the cross-correlation coefficients of IMF_3_ and IMF_4_ are substantially smaller than those in Table 4 and this indicates a very low correlation between them. The tendency of negative correlation values in Table 5 between IMFs across time scale is similar to those of in the (M, T) pairs. The relationship may also be observed from the plots in Fig 9. An enlarged view that highlights the difference in the local extrema and phase shift of 1-day cycle of the IMF curves is presented in Fig 10.

The value of correlation matrix (9, 9) in Table 5 is positive, this is the averaged value of all WSN nodes but individually, for example, as shown in Fig 9, it can be negative (i.e. -0.6). All the (M, P) cross-correlation coefficients calculated in 32 groups show that there are 19 positive values while other 13 values are negative. This may be an indication that the long-time relationship (more than 1000 hours) between soil moisture and PAR is unstable.

### 3.4 Spatial Correlation of Same Parameters

**Fig 11:**
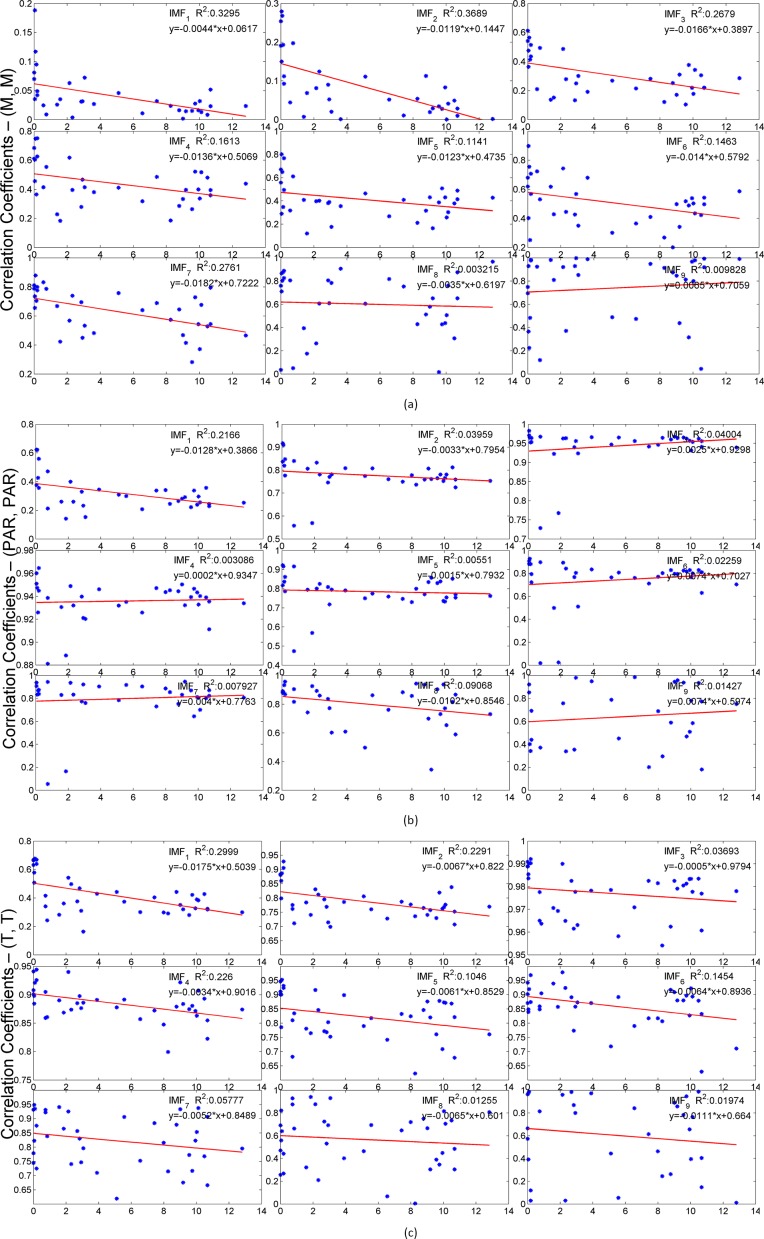
Cross-correlation Coefficients vs. Nodes’ Distance (Km)

In this subsection, we investigate how the environmental parameters measured by WSN nodes vary at different geographical locations. First we calculate each IMF’s Spearman’s rank cross-correlation coefficients for each of parameter pairs (M, M), (T, T) and (P, P) obtained from the corresponding WSN node pair described in Table 1. These parameter pairs include three categories: 1) the measurements from node pairs in the same subgroup, 2) the measurements among subgroups but in same group and 3) the measurements among the groups. We plot cross-correlation coefficients of each corresponding IMFs in each parameter pair (M, M), (T, T) and (P, P) versus its corresponding physical distance in Fig 11(A), 11(B) and 11(C) respectively.

From Fig 11(A) we can see that on relatively longer-time scale (IMF > = 3), two soil moisture measurements from two different geographical located WSN nodes always have higher correlation than those of in short-time scales (IMF < 3) regardless of their topographic distance. Spot distribution and linear regression line in the plots show that correlation coefficient values decrease as the two WSN nodes depart from each other. This may imply soil moisture’s correlation between two observation nodes will reduce while the distance between two observation nodes increases. When we inspect the correlation coefficient values in IMF_8_ and IMF_9_, little influence of distance on soil moisture’s correlation is observed on such long-time scales.

Fig 11(B) shows that the correlation between two temperature measurements at two different geographical located WSN nodes is always higher than that of soil moisture measurements, especially in 1-day circle (IMF_3_) where all correlation values are above 0.95. Linear regression lines and functions show that the distance has much less influence on land surface temperature than on those of soil moisture. Fig 11(C) also shows a similar conclusion. The reasons of these phenomena are because of the uniformity of land surface temperature and solar radiation in such a small and open field circumstance.

## 4. Conclusion

This paper presents a detailed analysis using the HHT based data analysis tools of the environmental data collected with a WSN, deployed in Yanqing, northwest of Beijing CBD in 2011, which provides medium regional-scale measurements of environment parameters for continuous crop field monitoring. The parameters include LST, soil moisture and solar radiation. We introduce the Hilbert-Huang transform as an empirical but adaptive tool to analyse the data set which was collected hourly over a three-month period. HHT based data analysis shows its ability to detect physically meaningful internal components and explain the spatial-temporal relationships among these environmental parameters. It provides new insights of the data with respect to the temporal and spatial variation of agricultural environment. Specifically,

1. This work shows strong localized spectrum information that enables abnormal values to be easily detected using EMD and EEMD techniques. Periodic information, especially the 24 hours cycle, is able to be extracted from data for all observed parameters.
2. Comprehensive correlation analysis is done by calculating the Spearman’s rank cross-correlation coefficients. Temporally, IMF plots illustrate that temperature is highly correlated with solar radiation (close to 0.8) and temperature is also correlated with soil moisture in an approximate diurnal cycle. We confirm from the IMF analysis that the above mentioned correlations are high for spatially widely separated locations. Spatial correlation of soil moisture is significant only when the sensed spots are closely spaced. In addition, IMF plots show a stable correlation between land surface temperature and solar radiation across the measured region.

It may be possible to obtain more interesting results if we use HHT to analyse longer environmental data sets when they are available. In addition to the intrinsic relationships among land surface temperature, soil moisture and solar radiation, the HHT based analysis may provide interesting view of the relationship between those mentioned parameters with respect to wind changes and reveal environmental changes as a result of global climate changes. Furthermore, machine learning methods have provided some solutions that achieved excellent performance in a wide variety of fields such as event detection, data-driven decision making and so on [4,19]. Supervised learning methods, e.g. Support Vector Machine (SVM) and Artificial Neural Network (ANN), use the labelled data (feature/label pairs) to build learning models and predict the unidentified instances according to these models. Thus, it should be interesting to combine the spatial-temporal relationships revealed in this paper with supervised learning methods to improve scenario analysis, sensor nodes location and precise measuring. These topics are considered in our ongoing research work.

## Supporting Information

S1 File Photon. This file includes the photon data of nodes 19 and 23.
(RAR)

S2 File Soil_moisture. This file includes the soil moisture data of nodes 19 and 23.
(RAR)

S3 File Temperature. This file includes the temperature data of nodes 19 and 23.
(RAR)
